# High Risk of Unexpected Late Fetal Death in Monochorionic Twins Despite Intensive Ultrasound Surveillance: A Cohort Study

**DOI:** 10.1371/journal.pmed.0020172

**Published:** 2005-06-28

**Authors:** Olivia Barigye, Lucia Pasquini, Paula Galea, Helen Chambers, Lucy Chappell, Nicholas M Fisk

**Affiliations:** 1Institute of Reproductive and Developmental Biology, Imperial College Londonand Centre for Fetal Care, Queen Charlotte's and Chelsea Hospital, LondonUnited Kingdom; **2**Perinatal Pathology Unit, Department of HistopathologyHammersmith Hospital, LondonUnited Kingdom; Kaplan Medical CentreIsrael

## Abstract

**Background:**

The rationale for fetal surveillance in monochorionic twin pregnancies is timely intervention to prevent the increased fetal/perinatal morbidity and mortality attributed to twin–twin transfusion syndrome and intrauterine growth restriction. We investigated the residual risk of fetal death after viability in otherwise uncomplicated monochorionic diamniotic twin pregnancies.

**Methods and Findings:**

We searched an electronic database of 480 completed monochorionic pregnancies that underwent fortnightly ultrasound surveillance in our tertiary referral fetal medicine service between 1992 and 2004. After excluding pregnancies with twin–twin transfusion syndrome, growth restriction, structural abnormalities, or twin reversed arterial perfusion sequence, and monoamniotic and high-order multiple pregnancies, we identified 151 uncomplicated monochorionic diamniotic twin pregnancies with normal growth, normal liquor volume, and normal Doppler studies on fortnightly ultrasound scans. Ten unexpected intrauterine deaths occurred in seven (4.6%) of 151 previously uncomplicated monochorionic diamniotic pregnancies, within 2 wk of a normal scan, at a median gestational age of 34^+1^ wk (weeks^+days^; range 28^+0^ to 36^+3^). Two of the five cases that underwent autopsy had features suggestive of acute late onset twin–twin transfusion syndrome, but no antenatal indicators of transfusional imbalance or growth restriction, either empirically or in a 1:3 gestation-matched case–control comparison. The prospective risk of unexpected antepartum stillbirth after 32 wk was 1/23 monochorionic diamniotic pregnancies (95% confidence interval 1/11 to 1/63).

**Conclusion:**

Despite intensive fetal surveillance, structurally normal monochorionic diamniotic twin pregnancies without TTTS or IUGR are complicated by a high rate of unexpected intrauterine death. This prospective risk of fetal death in otherwise uncomplicated monochorionic diamniotic pregnancies after 32 wk of gestation might be obviated by a policy of elective preterm delivery, which now warrants evaluation.

## Abstract 2

Despite intensive fetal surveillance, structurally normal monochorionic diamniotic twin pregnancies were found to be complicated by a high rate of unexpected intrauterine death late in pregnancy.

## Introduction

Monochorionic diamniotic (MCDA) twin placentation occurs in one in every 400 pregnancies, and is characterised by placental vascular anastomoses and thus interfetal transfusion [1]. MCDA twins are considered high risk by virtue of their 3- to 5-fold increased perinatal morbidity and mortality compared to dichorionic (DC) twins. This is largely attributed to twin–twin transfusion syndrome (TTTS), which occurs in 15%–20% of MCDA twin pregnancies, and discordant intrauterine growth restriction (IUGR), which complicates an additional 25% [1–5]. Further, in the event of intrauterine death (IUD) of one twin, there is a 40%–50% risk of death or neurological damage in the co-twin from acute intertwin transfusion [6–8]. There is also a minor contribution to the risk of MCDA twin pregnancies from a 2- to 4-fold increased risk of structural anomalies, particularly congenital heart disease [9,10]. With the decline in perinatal mortality in singletons in recent decades, increasing attention is now being given to improving outcomes in high-risk multiple pregnancies.

The rationale for heightened fetal surveillance in monochorionic (MC) compared to DC twins is early detection of TTTS and IUGR, to allow timely treatment in an attempt to prevent adverse perinatal outcome, principally by amnioreduction, endoscopic laser ablation of anastomoses, bipolar cord occlusion, or early delivery. Although the frequency of monitoring has not been evaluated in randomised trials for any type of twin pregnancy, it is common practice to monitor MC twins more frequently than DC twins. Despite a vigilant policy in our centre of fortnightly ultrasound surveillance for MCDA pregnancies, we observed that unexpected late fetal deaths still occurred in seemingly uncomplicated MCDA twins.

We aimed to determine the prospective gestational age-specific risk of unexpected IUD in uncomplicated MCDA twins after viability (24 wk of gestation). We also investigated the cause of such deaths and whether they could be predicted antenatally.

## Methods

We audited the perinatal outcome of all completed MC pregnancies seen over a 12-y period from 6 October 1992 to 31 August 2004, at the Centre for Fetal Care, Queen Charlotte's and Chelsea Hospital, a tertiary referral fetal medicine service in northwest London. Clinical details and ultrasound scan reports were retrieved from an electronic database (FileMaker Pro 5) of MC pregnancies, and augmented with case notes where necessary.

The term “uncomplicated” was used to denote pregnancies without features of TTTS on ultrasound that also had appropriate and concordant fetal growth, as well as normal growth velocity in each of two structurally normal twins. Appropriate growth was defined as an estimated fetal weight (EFW) greater than the fifth centile for gestational age [11]. The percentage EFW difference (ΔEFW) was calculated as the difference in weight, divided by the larger twin's weight, multiplied by 100, with growth defined as concordant if the ΔEFW was less than 25% [12]. Growth velocities were plotted against the charts of Chitty et al. [13–15]. The absence of TTTS in these pregnancies was denoted by normal and evenly distributed amniotic fluid volume (deepest vertical pool 2–8 cm in each sac) with concordant bladder dynamics [16]. These pregnancies also had normal umbilical artery (end diastolic frequencies present), umbilical vein (no pulsations), and/or ductus venosus (positive a wave) Doppler waveforms in each twin.

Uncomplicated MCDA pregnancies were monitored according to a standard protocol, which comprised routine first trimester nuchal translucency assessment and chorionicity determination, a detailed anomaly scan and fetal echocardiography at 20 wk, and then fortnightly scans for growth, amniotic fluid, and Doppler (umbilical artery, umbilical vein, and/or ductus venosus). Ductus venosus Doppler was added as a routine component in 1999 [16], and chorionic plate Doppler for compensatory artery-to-artery anastomosis (AAA) detection was added in 1995 [17]. Elective delivery was scheduled in otherwise uncomplicated pregnancies at between 36 and 37 wk of gestation.

We excluded pregnancies complicated by TTTS, IUGR, structural abnormalities, and twin reversed arterial perfusion, as well as high-order multiple, monoamniotic, and conjoined pregnancies. As this was a study of fetal death after viability, we also excluded pregnancies that delivered before 24 wk of gestation and those for which the pregnant woman returned to her local hospital for continued monitoring for geographical reasons. Six pregnancies with unavailable outcome data were also excluded.

The rate of fetal death in continuing uncomplicated MCDA pregnancies was derived for each 2-wk gestational block, beginning at 24 wk. It was calculated as the number of IUDs that occurred within the 2 wk following the beginning of week *n* divided by the number of continuing uncomplicated pregnancies at the beginning of week *n.* As we have previously applied to singleton pregnancies, the prospective risk of IUD was calculated as the total number of IUDs at or beyond the beginning of week *n* divided by the number of continuing pregnancies at or beyond the beginning of week *n* [18]. Prospective risk was not determined after 36 wk, as elective delivery resulted in too few continuing pregnancies beyond this time point.

To ensure that no features of IUGR or TTTS had been missed in pregnancies with an IUD, we compared antenatal indicators of these conditions at the last scan before the IUD in affected pregnancies with those in uncomplicated MCDA pregnancies unaffected by an IUD. The variables examined for IUGR were the abdominal circumference, head:abdominal circumference ratio, EFW, and ΔEFW. For TTTS, the amniotic fluid index (AFI) was used as the antenatal indicator; the deepest vertical pool of liquor in each twin's sac could not be compared, as they were not consistently recorded when the AFI was reported as normal and evenly distributed. The comparison used a 1:3 gestation-matched case versus control design (cases are pregnancies with an IUD; controls are those without). Variables in cases were from the last scan before the IUD; control scans were from the next three uncomplicated MCDA pregnancies appearing in the database for which the scan matched within 2 d of the case scan.

As the presence of an AAA reduces the risk of TTTS by a factor of four [17], and reduces severity in affected cases [19], we also compared the frequency of AAA detection by Doppler between cases and controls to determine whether functional AAAs were less common in pregnancies with an IUD; if so they would be more prone to develop TTTS than those unaffected by an IUD.

Finally, we reviewed the autopsy reports for any undetected pathology. In particular, we sought phenotypic features of TTTS such as organ hypertrophy, particularly cardiac hypertrophy in the larger twin.

For each variable in the 1:3 gestation-matched case–control comparison, we obtained three Δ values for every case by calculating the differences between the case and three control values**.** The mean and 95% confidence intervals (CIs) were then calculated for the Δ values (case variable minus control variable). Normally distributed variables were compared by one-sample *t* testing, and categoric variables by the Fisher exact test. Binomial CIs were calculated for proportions.

## Results

The total number of MC pregnancies during the period of study was 480, from which we excluded 164 with TTTS, 62 with IUGR, 27 with structural abnormalities, 21 monoamniotic pregnancies, 14 high-order multiples, nine with twin reversed arterial perfusion sequence, and two with conjoined twins. From the remaining 181 uncomplicated MCDA pregnancies, we excluded four that delivered before 24 wk, 20 referred back to their local hospitals for continued monitoring, and six for which delivery data were unavailable. The final study population was 151 apparently uncomplicated, intensively monitored MCDA twin pregnancies. Monochorionicity was the indication for referral in each case, and median maternal age was 32 y (range 16–43).

From this cohort of 151, there were ten unexpected fetal deaths in seven uncomplicated MCDA pregnancies (three double deaths and four single deaths) after 24 wk, giving an overall incidence of 4.6% per pregnancy (CI 1.9%–9.9%). Deaths occurred at a median gestation of 34^+1^ wk (range 28^+0^ to 36^+3^).

Table 1 shows the rate by 2-wk blocks of fetal death per pregnancy, which, with the limits of small numbers, appeared to increase after 32 wk. The prospective risk of unexpected fetal death, also detailed in Table 1, remained relatively stable between 24 and 34 wk of gestation, 1/22 (4.5%) and 1/30 (3.3%) pregnancies, respectively.

**Table 1:**
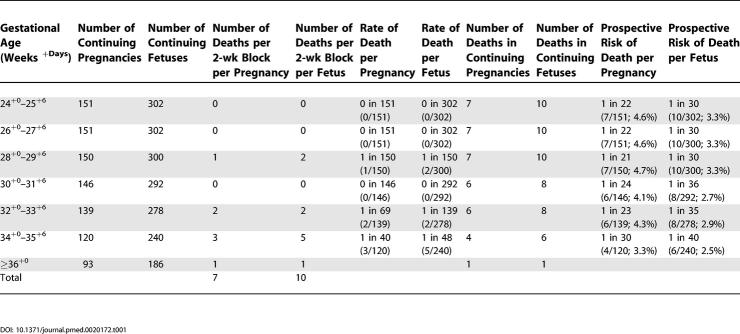
Rate and Prospective Risk of Unexpected Fetal Death in 151 Intensively Monitored, Uncomplicated MCDA Pregnancies after 24 wk of Gestation

DOI: 10.1371/journal.pmed.0020172.t001

Ultrasound findings on the last scan before IUD diagnosis and the clinical presentation at IUD are detailed in Table 2. Apart from one case that presented with reduced fetal movements, the finding of an IUD was incidental in six of the seven pregnancies at a routine scan. Growth was appropriate for gestational age and concordant, and the AFI was normal and evenly distributed in all seven affected pregnancies at the last scan prior to the diagnosis of IUD. All fetal deaths were diagnosed within 2 wk of a normal scan (median 1^+4^ wk, range 1^+0^ to 2^+0^).

**Table 2:**
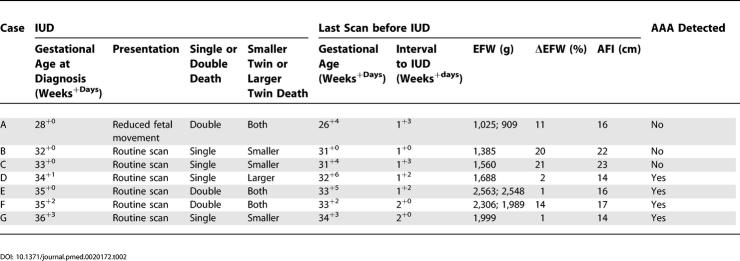
Antenatal Features of Pregnancies with an IUD (*n* = 7)

DOI: 10.1371/journal.pmed.0020172.t002

In the 1:3 gestation-matched case–control comparison, there was no significant difference between IUD-affected pregnancies and controls in any of the antenatal indicators of IUGR or TTTS. The mean Δ value for the abdominal circumference was 20 mm (CI −58 to +98), for the head:abdominal circumference ratio was −0.02 (CI −0.04 to 0.00), for EFW was −10 g (CI −142 to +122), for ΔEFW was −0.5% (CI −4.8 to +3.8), and for AFI was 1.2 cm (CI −1.4 to +3.8). AAA detection rates were similar in the two groups (54% in pregnancies with IUD and 60% in matched controls without an IUD). All the IUDs occurred in pregnancies monitored after the introduction of routine chorionic plate Doppler for AAA detection, as similarly did the selected 3:1 control pregnancies.

The post-mortem and placental findings are summarised in Table 3. After autopsy, deaths remained unexplained in three of five pregnancies (seven fetuses). However, two cases (A and F) had post-mortem evidence of TTTS, with the larger plethoric twins having cardiac hypertrophy, a feature that distinguishes TTTS from acute agonal intertwin transfusion, a common sequela of single IUD. In case F, a specific placental cause for sudden late onset TTTS was identified, both macroscopically and at histology, in the form of recent thrombosis in the recipient arterial component of a recipient–donor (compensatory) arteriovenous anastomosis. In case A, an AAA, which would otherwise decrease susceptibility to TTTS, was not detected antenatally nor on placental pathology, and hence the late onset of TTTS was unexplained. Both these double IUDs took place within 10–14 d of a normal scan, where the AFIs had been 16 and 17 cm and documented as evenly distributed, with fetal weight discordances of only 11% and 14%, respectively (Table 2). Thus, although these two double deaths could not be described as “unexplained” at autopsy, they were still unanticipated by serial fetal surveillance and thus “unexpected”.

**Table 3:**
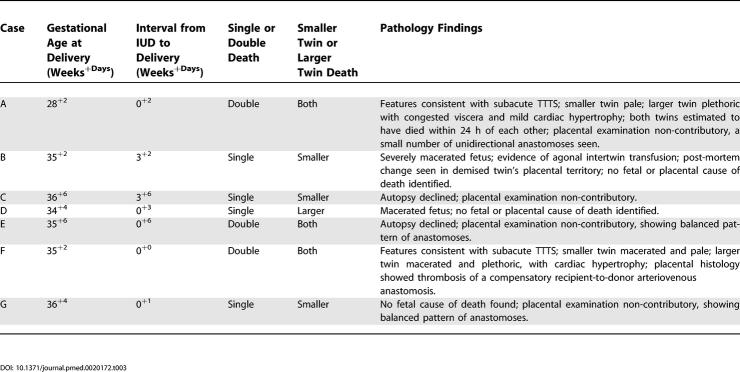
Autopsy and Placental Findings after IUD

DOI: 10.1371/journal.pmed.0020172.t003

## Discussion

We investigated the prospective risk of unexpected IUD in MCDA pregnancies with structurally normal fetuses, uncomplicated by TTTS or IUGR, which has not to our knowledge been reported in the literature to date. Population-based twin studies provide little information of relevance to this question, as they fail to stratify for chorionicity, fail to distinguish complicated from uncomplicated twin pregnancies, and record gestational age not at fetal death but at delivery, often many weeks later [20,21]. Indeed the prospective risk of stillbirth in twins overall has yet to be addressed in the literature. Older studies in both singletons and twins have concentrated on overall risks of fetal death as a proportion of total births, or weekly rates, rather than deriving prospective risks by gestation as a function of continuing pregnancies [22–24]. We have previously applied the latter approach to singletons to illustrate how prospective risk declines with gestational age and is a more useful measure both for patient counselling and timing of delivery, as it expresses the risk of fetal death at any particular point in gestation for the remainder of the pregnancy [18].

Sebire et al. suggested that the main risk of fetal death in MC pregnancies was before 24 wk, as after this, the rate of perinatal loss was only slightly higher in MC than in DC pregnancies (4.9% versus 2.8% of pregnancies) [3]. This, however, was a referral population with a high incidence of TTTS and other pathologies, and no recent ultrasound in many cases, and so is not comparable with our study of otherwise uncomplicated MCDA pregnancies which suffered an unexpected death within 2 wk of a normal scan.

Our data suggest instead that even intensively monitored, apparently healthy MCDA pregnancies remain at substantial risk of IUD after 24 wk (4.6% of pregnancies and 3.3% of fetuses). IUDs after 24 wk occurred in the third trimester, and predominantly after 32 wk of gestation, at which time the prospective risk of subsequent IUD was 1/23 pregnancies. Our findings provide useful information for counselling parents with MCDA pregnancies, and notwithstanding the limitation of small numbers, may be used to inform decisions regarding the optimal timing of delivery.

Analysis of the antenatal findings on fortnightly scans shows that deaths after 24 wk occurred unassociated with any antenatal evidence of either TTTS or IUGR, and thus were unexpected. Only two of the seven pregnancies complicated by an IUD had a definite cause at autopsy. Late onset TTTS is rare but not unknown, and here seems to have been unpredictable. Recent case reports [25,26] suggest that thrombosis of a previously patent compensatory anastomosis, either AAA or reverse arteriovenous, can result in haemodynamic imbalance and thus acute onset TTTS, as suggested in one of the two cases in this study. The fetal deaths in the present study occurred despite strategies aimed at preventing them, specifically, fortnightly ultrasound and Doppler surveillance in a tertiary fetal medicine unit, and elective delivery at 36–37 wk).

We acknowledge a number of limitations of our study. First, there is the retrospective nature of the analysis. Notwithstanding this, clinical and ultrasound findings were entered prospectively into our database, and we reviewed all cases entered over the 12-y period since its inception. Second, caution is advised in view of small numbers. However, this is the first series to our knowledge to report this outcome in otherwise normal MCDA twins, and the deaths were spread relatively evenly over the study period**.**Third, there is a lack of comparative data in other twin pregnancies. Use of a DC control group was precluded by they fact that our protocol for DC pregnancies differs from that for MCDA pregnancies, as in most centres, with DC monitoring performed outside the fetal medicine unit and at less frequent intervals (every 4 wk rather than every 2 wk), and with elective delivery 1 to 2 wk later than for MCDA pregnancies. In terms of complicated MC twin pregnancies, few with TTTS or IUGR and two live fetuses remain undelivered after 32 wk. We chose not to use the brain:liver ratio as an indicator of IUGR at post-mortem because of its insensitivity [27]. Birth weights were also not used as an indicator of discordant IUGR in view of the variable interval from death to diagnosis, and the even greater interval between death and delivery (up to several weeks) following single IUD, in which varying degrees of post-mortem weight loss and continued growth of the surviving twin might confound measured birth weights.

Our findings have a number of clinical implications. The high rate of unexpected third trimester fetal death might be obviated by a range of preventative strategies. One would be to increase the frequency of monitoring. Although growth is only usefully measured every 2 wk, more frequent surveillance could include amniotic fluid volume and distribution, and fetal Doppler waveforms. This might allow earlier identification of the occasional case of late onset TTTS, and thus prevention of IUD through timely delivery. However, it is not clear how more frequent monitoring would prevent unexplained IUDs, just as this strategy does not appear to prevent them in singleton pregnancies [28,29].

Another preventative approach might be earlier delivery. Complicated MCDA twin pregnancies, such as those with TTTS or abnormal umbilical artery Doppler, are already delivered in many centres at 32 wk. At this stage neonatal survival is now comparable to that at term [30,31]. Minor neonatal morbidity is still likely at 32 wk, but the chance of major respiratory and neurological problems is reduced substantially by administration of antenatal steroids [32]. Elective vaginal delivery would increase the chance of failed induction, but there is an increasing view that elective caesarean section is preferable to preterm induction of labour in MCDA twin pregnancies, both to avoid a caesarean section being performed as an emergency and to obviate the risks of acute intertwin transfusion during labour [33]. Accepting that elective delivery of all MC twins at 32 wk would carry an attendant neonatal morbidity, the complications of iatrogenic prematurity could be lessened if an intermediate gestation were chosen, e.g., 34 wk. Although neonatal morbidity would be reduced, so would the number of fetal deaths prevented. Acknowledging the limitation of small numbers in this study, 80% of fetal deaths at 32 wk or greater and 60% at 34 wk or greater, respectively, would have been prevented if fetuses were delivered before these gestation time points. When expressed per pregnancy, one case of IUD would be prevented for every 23 MC pregnancies delivered at 32 wk and one for every 30 pregnancies at 34 wk. The above figures underestimate the potential gain of such strategy, because single IUD in MCDA pregnancy also exposes the surviving co-twin to a substantial risk of brain injury and thus long-term handicap from acute intertwin transfusion [6–8].

Major neurodevelopmental sequelae are now rare in otherwise well babies delivered after 32 wk. MC twins are at increased risk of cerebral palsy compared to DC twins, but this is mainly prior to 32 wk and in pregnancies complicated by TTTS and IUGR [34,35]. Prematurity per se is not considered responsible for the high risk of neurological morbidity in MC twins, but rather the haemodynamic imbalance unique to MC placentation [36,37]. Thus, uncomplicated MC twins should not be at substantially increased risk of neurological sequelae from elective premature delivery at or after 32 wk. Indeed, elective delivery may reduce their risk of neurodevelopmental injury, as single IUD in MC twins is a well-established risk factor for cerebral palsy [6,38].

In conclusion, apparently uncomplicated MCDA pregnancies remain at substantial risk of fetal loss as they approach 32 wk of gestation, whereafter one in 23 (4.3%) will suffer an unexpected IUD. This risk is exacerbated by the 40%–50% risk of co-twin death or neurological injury following a single IUD [6]. If our findings are confirmed in other observational series, our suggestion that earlier delivery might prevent this adverse outcome could be tested by randomised trial.

**Patient Summary**

**Background**

As mothers wait longer to have children and more of them make use of reproductive technologies (medications that stimulate egg production and ripening, and in vitro fertilization, for example), multiple pregnancies (mostly twins and triplets) have become a lot more common. Such pregnancies carry a higher risk for mothers and fetuses, and obstetricians have developed more involved regimens of check-ups and tests to minimize those risks.

**Why Was This Study Done?**

Guidelines for how to best manage multiple pregnancies are still under development. This study looked at how the risk of complications changed from 24 weeks of pregnancy (the earliest time point after which preterm babies can survive) on throughout the last trimester.

**What Did the Researchers Do?**

They looked at identical (monozygotic) twins that shared a common placenta but had their own amniotic sac (the sac that holds the protective liquid called amniotic fluid that surrounds the fetus inside the uterus). Twins with these characteristics make up about two-thirds of all monozygotic twins. The researchers studied the records of one United Kingdom hospital that specializes in fetal care over a 12-year period (from 1992 to 2004) and looked at the clinical details and ultrasound scan records.

**What Did They Find?**

Of a total of 480 completed identical twin pregnancies with shared placentas, 151 were found to be uncomplicated; that is, both fetuses seemed normal and healthy on all of their regular check-up scans (done every two weeks). In seven of those 151 pregnancies, however, either one or both of the fetuses died late during pregnancy, without any prior warning signs.

**What Does This Mean?**

Despite intensive surveillance of women during pregnancy, this particular group of pregnant women experienced a substantial rate of unexpected fetal death. The numbers in this study are small, however, and this is only one study (one needs to be careful about drawing conclusions based on one small study). In addition, the study was not designed to test a specific question but just looked at the records retrospectively. For all of these reasons, it is not clear whether these results are representative of what happens in general.

**What Next?**

Larger studies need to test whether this risk of fetal death late in pregnancy among identical twins sharing a placenta is common. If follow-up studies confirm the preliminary results here, it might be worth delivering such twins prematurely (that is, at around 32 weeks of pregnancy), when the risks associated with premature birth are relatively small compared to the risk of losing one or both of the fetuses after that date.

**Additional Online Resources**

Information about different types of multiple pregnancies, screening guidelines, and possible complications can be found at the following sources.

OMNI (Organising Medical Networked Information) Web page on multiple pregnancy: http://omni.ac.uk/browse/mesh/D011272.html

An editorial from the *Medical Journal of Australia:* http://www.mja.com.au/public/issues/178_12_160603/ums10064_fm-2.html

Web page from the Health on the Net Foundation: http://www.hon.ch/Dossier/MotherChild/complications/complicate_multiple.html

Web Page from the University of Maryland Medical Center: http://www.umm.edu/pregnancy/specialcare/articles/multipreg.html

## Abbreviations

ΔEFW: percentage estimated fetal weight difference
AAA: artery-to-artery anastomosis
AFI: amniotic fluid index
CI: 95% confidence interval
DC: dichorionic
EFW: estimated fetal weight
IUD: intrauterine death
IUGR: intrauterine growth restriction
MC: monochorionic
MCDA: monochorionic diamniotic
TTTS: twin–twin transfusion syndrome
